# Bridging-to-Surgery in Patients with Type 2 Intestinal Failure

**DOI:** 10.1007/s11605-020-04741-0

**Published:** 2020-07-22

**Authors:** Fleur E. E. de Vries, Jeroen J. M. Claessen, Elina M. S. van Hasselt-Gooijer, Oddeke van Ruler, Cora Jonkers, Wanda Kuin, Irene van Arum, G. Miriam van der Werf, Mireille J. Serlie, Marja A. Boermeester

**Affiliations:** 1 Department of Surgery, Amsterdam University Medical Centers, location AMC, Postbox 22660, 1100 DD Amsterdam, The Netherlands; 2 Department of Surgery, IJsselland Ziekenhuis, Capelle a/d IJssel, The Netherlands; 3 Nutrition Support Team, Amsterdam University Medical Centers, location AMC, Amsterdam, The Netherlands; 4 Department of Endocrinology and Metabolism, Amsterdam University Medical Centers, location AMC, Amsterdam, The Netherlands

**Keywords:** Intestinal failure type 2, bridging-to-surgery, parenteral nutrition, high-output, fistula, stoma

## Abstract

**Aim:**

Type 2 intestinal failure (IF) is characterized by the need for longer-term parenteral nutrition (PN). During this so**-**called bridging-to-surgery period, morbidity and mortality rates are high. This study aimed to evaluate to what extent a multidisciplinary IF team is capable to safely guide patients towards reconstructive surgery.

**Methods:**

A consecutive series of patients with type 2 IF followed up by a specialized IF team between January 1st, 2011, and March 1st, 2016, was analyzed. Data on their first outpatient clinic visit (T1) and their last visit before reconstructive surgery (T2) was collected. The primary outcome was a combined endpoint of a patient being able to recover at home, have (partial) oral intake, and a normal albumin level (> 35 g/L) before surgery.

**Results:**

Ninety-three patients were included. The median number of previous abdominal procedures was 4. At T2 (last visit prior to reconstructive surgery), significantly more patients met the combined primary endpoint compared with T1 (first IF team consultation) (66.7% vs. 28.0% (*p* < 0.0001), respectively); 86% had home PN. During “bridging-to-surgery,” acute hospitalization rate was 40.9% and acute surgery was 4.3%. Postoperatively, 44.1% experienced a major complication, 5.4% had a fistula, and in-hospital mortality was 6.5%. Of the cohort, 86% regained enteral autonomy, and when excluding in-hospital mortality and incomplete follow-up, this was 94.1%. An albumin level < 35 g/L at T2 and weight loss of > 10% at T2 compared with preadmission weight were significant risk factors for major complications.

**Conclusion:**

Bridging-to-surgery of type 2 IF patients under the guidance of an IF team resulted in the majority of patients being managed at home, having oral intake, and restored albumin levels prior to reconstructive surgery compared with their first IF consultation.

**Electronic supplementary material:**

The online version of this article (10.1007/s11605-020-04741-0) contains supplementary material, which is available to authorized users.

## Introduction

Intestinal failure (IF) is defined as a reduction in functional capacity below the minimum necessary for sufficient absorption of macro- and micronutrients and/or fluids and electrolytes. Intravenous supplementation is required to maintain health and/or growth.^1^ IF can occur after an abdominal catastrophe resulting in, for example, a high-output stoma, an enterocutaneous fistula (ECF), or an enteroatmospheric fistula (EAF).

IF^1^ is categorized into three types with different treatment options and prognoses. Type 1 is reversible and self-limiting IF, whereas type 3 is chronic and mostly irreversible. Type 2, however, can be reversible and is characterized by the temporary need for parenteral nutrition (PN) and/or fluids for several months until reconstructive surgery. During this so**-**called bridging-to-surgery period, many patients are metabolically unstable and hospitalized. Consequently, morbidity and mortality are high.^2^–^4^

Nowadays, most specialized centers recommend postponing reconstructive surgery for at least 6 months, whereas in the past, reconstructive surgery was performed within a few weeks.^1^ By delaying surgery, abdominal infections can be managed and patients can recover to nutritional, physical**,** and mental health. Not all surgeons are aware of the importance of this waiting period as type 2 IF is relatively rare. As hospitalization is frequent and morbidity is high, most surgeons feel the urge to reconstruct the bowel**,** and many times patients are rushed into surgery. Under very strict monitoring of weight; diet; nutrient-, fluid-, and electrolyte balances; specialized home-care and regular outpatient clinic visits that are supervised and managed by a specialized and dedicated multidisciplinary IF team, patients may be able to recover before reconstructive surgery in their home situation and receiving PN. In 2016, the European Society of Coloproctology (ESCP) intestinal failure consensus statement^1^ was published. This statement, based on a systematic literature review and modified Delphi process, was written by 15 IF specialists in Europe. They recommend that type 2 IF should be treated in a multidisciplinary IF unit. In Table 1 we summarize important treatment principles for the “bridging-to-surgery” period for patients with type 2 IF as derived from the ESCP consensus statement.

**Table 1:** Treatment principles in the bridging-to-surgery period derived from the ESCP consensus statement^1^

Recommendation	Level of evidence
Type 2 IF (>28 days) should be treated in a multidisciplinary IF unit	4
Reconstructive surgery should not be undertaken for 6–12 months and until nutrition has been optimized, and preferably after a patient has had a period of time at home. A few parameters of optimizing are rising albumin levels (preferably >32 g/L), resolution of sepsis, good fluid and electrolyte balance, and stable or increasing weight.	4
Patients should be allowed to take liquids and diet as early as possible and as tolerated unless the surgeon feels that withholding oral intake may reduce peritoneal contamination and provide the best chance of spontaneous closure immediately after fistula formation	5
Specific nutrient deficiencies need to be monitored with regular measurements of magnesium, zinc, selenium, iron, vitamins D, K, B_12_ in those requiring prolonged nutritional support, particularly if there are difficulties with oral magnesium and phosphate supplementation with a high-output stoma/fistula.	4
High-dose loperamide, proton pump inhibitors, and codeine phosphate should be used to reduce fistula or stoma output. There is little evidence to support the routine use of somatostatin analogues or cholestyramine in the management of high output stoma or intestinal fistula.	4

This study aimed to evaluate the effect of treatment of type 2 IF patients who underwent reconstructive surgery by a specialized multidisciplinary intestinal failure team on pre-operative and postoperative outcomes and thereby sharing our knowledge and experiences.

## Methods

The manuscript was written in accordance with the Strengthening the Reporting of Observational Studies in Epidemiology (STROBE) statement.^5^

### Study Design and Patient Inclusion

This retrospective cohort study was performed in the Amsterdam University Medical Centers (UMC), location AMC, the national tertiary referral center for type 2 IF in Amsterdam, the Netherlands. A consecutive series of patients followed up by a specialized IF team between January 1st**,** 2011, and March 1st**,** 2016, was screened for eligibility. The study was approved by the Medical Ethical Committee of our hospital and the need for informed consent was waived due to the observational study design.

All patients referred to the outpatient clinic of the IF unit with acute intestinal failure, PN for more than 28 days, and who eventually underwent reconstructive surgery within the inclusion period were included (type 2 IF). Referrals consisted of patients that were already on home PN and as such already discharged from their initial admission or visited the outpatient clinic by ambulance from the referral hospital or rehabilitation center. Patients with (prolonged) type 1 or type 3 IF, patients with a single visit for a second opinion**,** and patients with incomplete documentation were excluded from the study.

### Treatment Principles for Patients with Type 2 Intestinal Failure

In short, treatment was according to the SNAP (sepsis, nutrition, anatomy, plan) principles^6^ and our updated in-house protocol^7^, which is added as Text S1. All patients were treated following our algorithm of standard care (Figure S1) consisting of medication to reduce output, an oral isotonic fluid restriction of 1 liter**,** and a short bowel diet according to the ESPEN guidelines.^8^

### IF Team

The IF team consisted of an intestinal failure surgeon, an internist-endocrinologist, physician assistant, (IF) specialized nurses, and dietitians. IF outpatient care was organized as a “one-stop” multidisciplinary consultation, during which a patient was seen at 4-, 8-, or 12-week intervals by all team members to discuss general wellbeing/performance status, nutritional state, lab results, medication, PN and/or intravenous fluid administration, fistula or stoma output, inspection of fistula/wound/central venous catheter (CVC), and rehabilitation status. Based on the outcome of these items, a treatment plan was made for the upcoming period. Twice a month, patients had contact with the physician assistant/specialized IF nurse and dietitian per e-mail or telephone to follow up on treatment and monitor lab results, medication, weight, oral intake, stoma/fistula output, CVC, amount of administered PN and/or intravenous fluid which could be adjusted if necessary. Specialized home-care teams, responsible for the administration of intravenous fluid and/or PN and medication, were instructed to follow the treatment plan and contacted the IF team directly if deemed necessary.

Plastic/reconstructive surgeons were consulted in IF cases of large full-thickness skin defects or in patients with abdominal wall defects associated with significant loss of domain and need of abdominoplasty; these reconstructions are a joined effort of the plastic surgeon and IF surgeon.

### Data Collection and Outcome Measures

Data were collected retrospectively using the electronic patient record system, and by checking patient files (retrieved from the referring hospitals). Data was collected on the first outpatient clinic visit (T1), the last outpatient clinic visit before reconstructive surgery (T2), emergency visits during the “bridging-to-surgery” period, and postoperative outcomes.

The included data consisted of (baseline) patient characteristics, past abdominal surgeries, nutrition, laboratory results, surgery reports, and clinical outcomes.

### Outcomes

The primary outcome was the composite endpoint of recovery at home (partial), oral/enteral intake**,** and a normal albumin level (> 35 g/L) at T2 (last visit prior to surgery) compared with T1 (first IF team consultation) in patients having reconstructive surgery. Secondary outcomes included living situation, oral intake, albumin, body weight, body mass index (BMI), stoma or fistula output, the amount of PN**,** and intravenous fluids in milliliters during 7 days prior to visits T1 and T2, and time to reconstructive surgery. Adverse events (abscess drainage, emergency department visits, acute hospitalization, acute surgery, and CVC**-**related complications) during the “bridging-to-surgery” period were assessed. Postoperative outcome measurements were based on the consensus statement by the ESCP intestinal failure group^1^ and included 30-day and in-hospital mortality, unplanned reoperation, unplanned hospital readmission < 30 days, recurrent fistulation, and the ability to discontinue TPN after 2 years of follow-up. Clavien–Dindo ≥ grade 3 complications and long-term mortality were also recorded.

### Definitions

The number of abdominal operations was defined as the number of abdominal interventions in an operation room where the abdominal cavity was entered. The difference in weight and BMI was defined as the difference (in percentages) between the preadmission body weight (i.e., before the first major abdominal surgery leading to IF) and the weight or BMI at respectively T1 and T2. Oral and/or enteral nutrition was defined as any amount of solid or liquid food intake. High output was defined as stoma output more than 1500 milliliters (ml) per day and fistula output more than 500 milliliters (ml) per day.^1^, ^9^ The quantity of PN and parenteral fluid administration was registered as the total PN and parenteral fluid administration in milliliters per week as not all patients received PN or parenteral fluids daily. CVC**-**related infection was defined by the presence of positive blood cultures in the absence of another infectious focus. Catheter**-**related thrombosis was defined as the presence of a thrombus proven by ultrasonography or phlebography. Regained intestinal autonomy was defined as the discontinuation of both PN, parenteral fluids**,** and electrolyte administration, after reconstructive surgery. For patients who were still within a 2-year follow-up period of intestinal adaptation, the most recent available data on their physical health and nutritional state was used.

### Statistical Analysis

Statistical analyses were performed with SPSS version 23.0 (IBM Corp. Released 2012. IBM SPSS Statistics for Windows, Version 23.0. Armonk, NY: IBM Corp). Descriptive statistics were performed to determine frequencies and percentages within cases. The mean (including standard deviation) was used in data with a normal distribution and the median (including interquartile range (IQR) or range) was used in data that did not have a normal distribution. The McNemar test was used on paired nominal data. The Wilcoxon signed-rank test was used on paired not normally distributed data. To determine the risk factors for morbidity and in-hospital mortality, significant variables were identified using univariate analysis. Categorical variables were compared using Fisher’s exact test. All comparisons were two-tailed probabilities. A *p* value ≤ 0.05 was considered statistically significant.

## Results

A total of 120 out of 264 consecutive patients who visited the IF outpatient clinic between January 2011 and March 2016 met the inclusion criteria. Another 27 patients were excluded since they did not have reconstructive surgery for the following reasons; managed non-operatively (*n* = 7), still within “bridging-to-surgery” period (*n* = 5), died before reconstructive surgery (*n* = 9), and no surgical options (*n* = 6). Finally, 93 patients were enrolled in the analyses. Figure S2 depicts the flow chart of patient inclusion.

### Patient Characteristics

Baseline characteristics are presented in Table 2. The median age was 64 (range 26–83) years and 50% was female. Most patients were referred from other, mostly regional hospitals. The majority of patients (57%) had an ECF or enteroatmospheric fistula (EAF, with visible bowel mucosa). Sixty-nine patients (74.2%) had a high-output stoma of high-output fistula. The median number of previous abdominal operations was 4 (IQR 3-7), with one patient having 24 previous abdominal procedures.

**Table 2:** Baseline characteristics

	No. of patients (*n* = 93)
Age in years (median, range)	64 (26–83)
Gender (male:female)	47 : 46
Preadmission body mass index (BMI) (median, IQR)	25.5 (22.6–29.2)
Referral	
Internal External	15 (16.1%) 78 (83.9%)
Time between last abdominal intervention and first visit (T1) in months (median, IQR)	2 (1-5)
Diabetes Mellitus	17 (18.3%)
Smoking	17 (18.3%)
IBD	18 (19.4%)
Immunosuppressive medication	8 (8.6%)
Number of previous abdominal operations (median, IQR)	4 (3–7)
Etiology type 2 intestinal failure	
ECF# Anastomotic leakage Perforation iatrogenic Mesh related Perforation inflammatory / infection± Ischemic bowel Other^ EAF* after open abdomen treatment High output enterostomy Anastomotic leakage Perforation iatrogenic Perforation inflammatory/infection± Ischemic bowel Oncologic resection Radiation enteritis	39 (41.9%) 15 (16.1%) 9 (9.7%) 5 (5.4%) 5 (5.4%) 1 (1.1%) 4 (4.2%) 15 (16.1%) 37 (39.8%) 9 (9.7%) 1 (1.1%) 7 (7.5%) 19 (20.4%) 1 (1.1%) 2 (2.2%)
High-output fistula or stoma	69 (74.2%)
Small bowel length to enterostomy/fistula	
< 50 cm 50–100 cm 100–150 cm 150–200 cm 200–250 cm > 250 cm	3 (3.2%) 9 (9.7%) 16 (17.2%) 9 (9.7%) 5 (5.4%) 51 (54.8%)
Colon length	
< Hemicolon in situ Ileocaecal valve in situ	35 (37.6%) 56 (60.2%)
Central catheter at first visit	
None CVC^1^ PICC^2^ Port-a-cath	2 (2.1%) 55 (59.2%) 34 (36.6%) 2 (2.1%)

^#^Enterocutaneous fistula

^±^Inflammatory bowel disease/pancreatitis/diverticulitis

^^^After open abdomen treatment/oncologic resection/unknown

*Enteroatmospheric fistula

^1^Central venous catheter

^2^Peripheral inserted central catheter

### Primary Outcome

The median period between the last abdominal intervention and reconstructive surgery was 9 months (IQR 7–11). When comparing T1 (first IF team consultation) to T2 (last visit prior to reconstructive surgery), a significant increase was seen in the proportion of patients that had reached the combined primary endpoint, 28.0% vs. 66.7% (*p* < 0.0001; Table 3).

**Table 3:** “Bridging-to-surgery” period, comparison of T1 (first visit) and T2 (pre-operative visit)

	First visit (T1)*	Pre-operative (T2)*	*p* value
Primary outcome$	26 (28.0%)	62 (66.7%)	<0.0001
Living situation			
At home Hospital Rehabilitation home	53 (57.0%) 33 (35.5%) 7 (7.5%)	79 (84.9%) 8 (8.6%) 6 (6.5%)	< 0.0001 < 0.001 0.220
Oral intake	59 (63.4%)	87 (93.5%)	< 0.0001
Albumin > 35 g/L	51 (54.8%)	71 (76.3%)	< 0.0001
Weight loss (kg)^1^	6.5 (IQR 1-12)	3 (IQR 0-8)	< 0.0001
BMI (kg/m^2^)	23.2 (IQR 20.5-26.4)	24.2 (IQR 22.0-27.1)	< 0.0001
Parenteral administration			
Patients with only PN PN administration per week (mL) Patients with only fluids Fluid administration per week (mL) Patients with PN and fluids PN administration per week (mL) Fluid administration per week (mL)	36 (38.7%) 14000 (11944–14000) 6 (6.5%) 10500 (7000–17500) 44 (47.3%) 12250 (10500–14000) 7000 (5688–10500)	13 (14.0%) 11760 (10500–14000) 11 (11.8%) 10500 (7000–14000) 56 (60.2%) 10500 (7000–14000) 7000 (5250–10500)	
Medication			
PPI Loperamide Somatostatin analogue Codeine	73 (78.5%) 40 (43.0%) 15 (16.1%) 20 (21.5%)	78 (83.9%) 48 (51.6%) 16 (17.2%) 15 (16.1%)	

*Values in median (IQR)

^$^Primary outcome = living situation at home + oral intake + albumin > 35 g/L

^1^Compared with preadmission body weight

### Secondary Outcomes

**Fig. 1:**
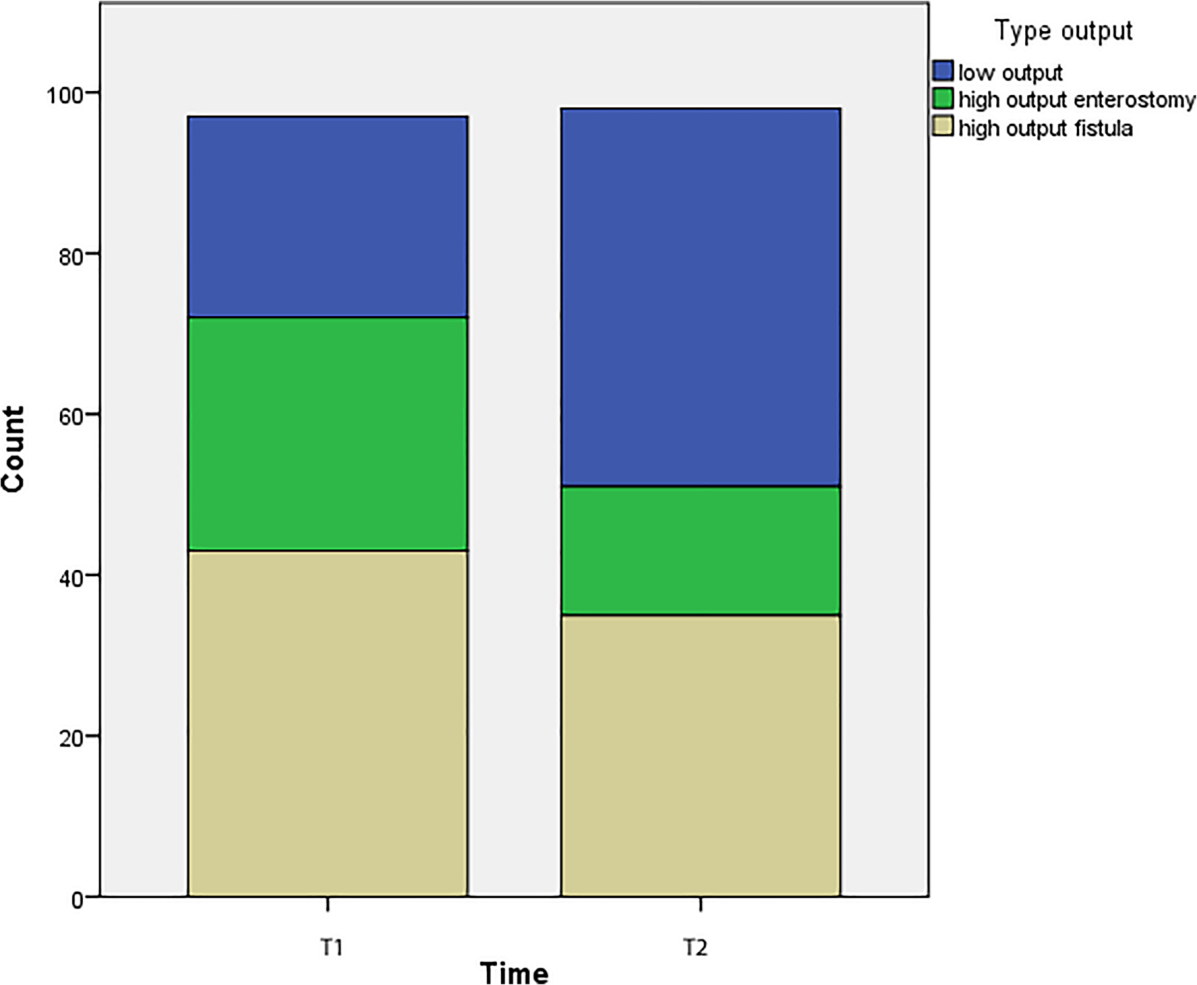
Counts of patients with low- and high-output fistula or enterostomy at T1 compared with those at T2

All individual parameters of the primary outcome also showed a significant increase when analyzed separately. Only 8.6% of patients were hospitalized at the time of reconstructive surgery, and 93.5% were able to tolerate oral/enteral nutrition. Median weight loss improved from 6.5 kg (IQR 1–12) at the first visit (T1) to 3 kg (IQR 0–8) before reconstructive surgery (T2). Compared with T1, at T2, more patients received a combination of PN and parenteral fluids. The amount of PN was lower at T2 explained by the higher oral intake at T2. All secondary outcomes are presented in Table 3. As shown in Fig. 1, at T1 74% of the patients had a high-output fistula or enterostomy compared with 50% at T2 (*p* = 0.018).

### Adverse Events During the “Bridging-to-Surgery” Period

During the “bridging-to-surgery” period**,** 31 patients (33.3%) visited the emergency department at least once. The majority of patients presented with fever, abdominal pain, dehydration**,** or stoma-related problems. Thirty-eight patients (40.9%) were admitted to the hospital and eight patients (8.6%) needed abscess drainage. Four patients (4.3%) underwent emergency surgery. The reasons for emergency surgery were ischemic prolapse of an ECF, suspicion of a strangulated parastomal herniation, sepsis due to persistent fistula formation and abdominal retentions**,** and one patient presenting with an ileus requiring adhesiolysis. Catheter**-**related complications occurred in 18 patients (19.4%), most were CVC-related infections (16 / 18) (Table 4).

**Table 4:** Adverse events during “bridging-to-surgery”

Event	No. of patients (*n* = 93)
Emergency department visit	
No Yes, Amsterdam UMC Yes, other hospitals	62 (66.7%) 25 (26.9%) 6 (6.4%)
Unplanned hospitalization	
No Yes, Amsterdam UMC Yes, other hospitals	55 (59.1%) 25 (26.9%) 13 (14.0%)
Abdominal abscess drainage	8 (8.6%)
Unplanned surgery	4 (4.3%)
Catheter-related complications Infection Thrombosis	18 (19.4) 16* 2^

*14 with a central venous catheter and 2 with a PICC line

^1 patients with a central venous catheter and 1 patient with a PICC

### Reconstructive Surgery

Operative details of the reconstructive surgery are presented in Table 5. The procedure was performed in the Amsterdam UMC, location AMC, in 68 patients (73.1%), and in referring hospitals in 25 (26.9%). When the operation took place in the referring hospital, the specialized surgeon of the IF team of the Amsterdam UMC was present in most of the cases.

**Table 5:** Operative details

	No. of patients (*n* = 93)
Number of anastomoses (median, range)	1 (0–4)
Resection of one or more enteric fistulas	59 (63.4%)
Restoration of continuity	59 (63.4%)
Removal of synthetic mesh	9 (9.7%)
Component separation technique performed Unilateral Bilateral	45 (48.4%) 6 (13.3%) 39 (86.7%)
Primary fascial closure achieved	81 (87.1%)
The use of IPOM biologic mesh Reinforcement Bridging	49 (52.7%) 37 (75.5%) 12 (24.5%)
Stoma takedown	37* (90.2%)

Values in parentheses are percentages unless indicated otherwise

*IPOM*, intra-peritoneal onlay mesh

^of 41 with enterostomy

Restoration of continuity was performed in 59 patients (63.4%). In 59 patients (63.4%), one or more enteric fistulae were resected. A synthetic mesh was removed in 9 patients (9.7%). Fascial closure was achieved in 81 patients (87.1%). A non-cross-linked biological mesh (Strattice^TM^) was used in 49 patients (52.7%) and in 37 patients as reinforcement with primary fascial closure and in 12 patients as bridging technique when primary fascial closure was not possible.

### Postoperative Outcomes

All postoperative data are presented in Table 6. Thirty-day mortality was 2.2% (2 patients) and in-hospital mortality was 6.5%. The cause of death was abdominal septic complications (*N* = 3) or due to an underlying disease (*N* = 3; cardiac and renal failure, respiratory failure**,** and a newly discovered metastatic esophagus carcinoma). A total of 41 patients (44.1%) experienced a ≥ grade 3 Clavien–Dindo grade complication. Six patients underwent an unplanned reoperation and 5.4% had a postoperative fistula, all were recurrent fistula. Four of these five patients underwent a successful second reconstructive surgery, and one other patient has a stable very low-output fistula which is managed non-operatively. In all other patients with fistulas, restoration of continuity was successful. During follow-up, two patients died before discontinuation of TPN.

**Table 6:** Postoperative outcomes

	No. of patients (*n* = 93)
Time between first contact (referral) and reconstructive surgery in months (median, IQR)	5 (4–7.5)
Time between last abdominal intervention and reconstructive surgery in months (median, IQR)	9 (7–11)
30-day mortality In-hospital mortality	2 (2.2%) 6 (6.5%)
Unplanned reoperation after reconstructive surgery for type 2 intestinal failure < 30 days	6 (6.5%)
Clavien–Dindo classification grade 3–4 complications	41 (44.1%)
Postoperative fistulas (all recurrent)	5 (5.4%)
Unplanned hospital readmission < 30 days after discharge	11 (11.8%)
Intravenous supplementation dependency after 2-year follow-up	
None PN Fluid PN + fluid	80 (86.0%) 5 (5.4%) 1 (1.1%) 7 (7.5%)
Reason postoperative TPN/fluid administration§	
Unable to discontinue PN/fluid Chronic PN dependence Chronic fluid dependence Died before discontinuation Not completed follow-up (postoperative period < 2 years)	2 (2.2%) 1 (1.1%) 8 (8.6%) 2 (15.4%)
Long-term mortality Days after reconstructive surgery (median, IQR)	11 (11.8%) 246 (46–525)

^§^*n* = 13

At 2 years of follow-up (*N* = 85), only 5 patients were unable to discontinue PN while enteral autonomy was regained in 94% (80/85).

### Risk Factors for Poor Operative Outcome

Univariate analysis showed that an albumin level < 35 g/L at T2 was a significant risk factor for ≥ grade 3 Clavien–Dindo complications, whereas a weight loss ≥ 10% at T2 compared with preadmission body weight was a significant risk factor for in-hospital mortality (Table 7).

**Table 7:** Univariate analysis of risk factors for ≥ grade 3 Clavien-Dindo complications and in-hospital mortality

Risk factors	Complications CD grade ≥ 3			In-hospital mortality		
	< CD3	≥ CD3	*p*	Alive	Death	*p*
Sex			0.537			0.435
Female Male	26 26	20 21		42 45	4 2	
External referral			1.00			0.585
Yes No	8 44	7 34		15 72	6 0	
Diabetes			0.794			0.588
Yes No	9 43	8 33		17 70	0 6	
Active smoking			1.00			1.00
Yes No	7 45	6 35		75 12	1 5	
Etiology fistula			0.297			0.696
Yes No	27 25	26 15		49 38	4 2	
IBD			0.186			0.592
Yes No	13 39	5 36		18 69	0 6	
Immunosuppression			0.459			1.00
Yes No	6 46	2 39		6 79	0 6	
Living situation at home at T2			0.144			0.221
Yes No	47 5	32 9		75 12	4 2	
Oral intake at T2			0.400			0.388
Yes No	50 2	37 4		82 5	5 1	
Weight loss > 10% still present at T2			0.341			*0.038*
Yes No	11 40	13 28		20 66	4 2	
High output at T2			0.529			0.205
Yes No	24 26	23 18		42 43	5 1	
Albumin< 35 g/L at T2			*0.016*			0.597
Yes No	5 44	13 27		4 67	2 4	

Italicized numbers are significant *p* values

## Discussion

We here show that intensive management by a specialized multidisciplinary IF team during the “bridging-to-reconstructive surgery” period in type 2 IF patients resulted in a significantly higher proportion of patients reaching the composite primary endpoint (recovery at home, partial oral/enteral food intake, and normal albumin (> 35 g/L)) prior to their planned surgical reconstruction compared with their first IF team consultation (*p* < 0.0001). The number of adverse events during the “bridging-to-surgery” period was considerable and can be explained by this specific type of seriously ill patients. Postoperative outcomes are comparable with other series in literature.^10^–^13^

This consecutive series of patients have had at least 28 days of PN from the beginning of their abdominal catastrophe and according to the EPSN guidelines are IF type 2 patients. We excluded patients that were on chronic PN and in which IF is not reversible. Furthermore, we wanted to share our knowledge and experiences to what end our treatment effects also postoperative outcomes. Therefore, we included only patients that underwent reconstructive surgery and we excluded not only deceased patients, in which treatment failed, but also patients that were managed non-operatively and where treatment was successful. Excluding these patients could lead to selection bias, but that is also our intent since we wanted to evaluate our approach and methods in a consecutive cohort of patients that ultimately underwent reconstructive surgery. This does not reflect all IF patients since type 2 IF patients are difficult to manage as they have no stable disease yet. Type 2 IF patients can still be catabolic, metabolically unstable, and usually have complex abdominal wounds and fistulas. Our approach differs from most other centers in the Netherlands and Europe in that we manage type 2 IF patients at home, thereby reducing admission time after the most acute infectious problems are managed, while in most countries, patients are hospitalized until reconstruction surgery or cessation of PN. This may even lead to unwanted shortening of the necessary “bridging to surgery” time, thereby increasing complication risk

For such ill patients with high output stomas or fistulas and type 2 IF, being able to recover at home is deemed impossible for most patients and surgeons. With home-care facilities and close monitoring of dietary, fluid, nutritional**,** and vital parameters, we managed to send the majority of these patients home safely. Hospitalization and especially immobilization can affect both physical and mental condition.^14^ Only eight patients were not able to leave the hospital and six patients recovered in a rehabilitation home. This was mostly due to pre-existent conditions such as social reasons or unstable mental health making home-care PN too complex or dangerous.

A nihil per os (NPO) policy is only necessary in a very specific subset of patients. However, NPO instructions are still often mistakenly prescribed by clinicians. Often the motivation is to increase chances of spontaneous closure of ECF/EAF. However, if fistula closure does not occur in the first 6 weeks, spontaneous closure is very unlikely. Moreover, EAF never close spontaneously and high-output fistula are known for their low closure rate.^2^, ^15^ Therefore, patients should be allowed to take oral fluids and diets as early as possible^1^, unless a real closure opportunity is expected. Another reason to maintain a patient on NPO can be an uncontrollable high-output fistula or enterostomy. Although sometimes a real challenge, with dedicated wound and stoma care nurses and the right medication and materials, almost all ECF can become controllable. In our cohort**,** 37% were on an NPO regime upon referral and oral intake increased from 63 to 93% without comprising fluid or electrolyte balance. This indicates that it is safe to have at least some oral intake of food under strict supervision of output, dehydration**,** and electrolyte disturbances. In our experience, oral intake is important for quality of life and improves mental recovery. Moreover, feeding the gut preserves mucosa integrity^16^, ^17^ and might improve intestinal adaptation postoperatively.

We included albumin in our primary endpoint. Although the idea that albumin is correlated with nutritional status has been abandoned, extensive literature^18^–^21^ shows a significant association between low albumin levels and postoperative morbidity, especially fistula recurrence and surgical site infections^2^ and mortality. In our cohort, low albumin was found to be a risk factor for ≥ grade 3 Clavien–Dindo complications. This might be explained by the fact that low albumin reflects an ongoing inflammatory status.^21^ The definition of low albumin differs in literature. We chose for ≤ 35 g/L as this is used as the cut-off value in our hospital and often used in literature.^21^ However, the ESCP guidelines^1^ mention preferably > 32 g/L. We initially also aimed to investigate other blood parameters. However, as most deficiencies (especially magnesium and phosphate) were already corrected by the referral hospitals and are included in PN, a longitudinal prospective study with several consecutive time points and details about supplementation treatment is needed to draw meaningful conclusions.

An interesting finding is that most of the patients did not receive enough intravenous fluid supplementation at their first referral visit. As in high-output fistula or enterostomies, excessive fluid losses will cause thirst, a logical response is to increase oral intake (usually water). However, patients with type 2 IF have a functional short-bowel with insufficient capacity to absorb high volumes of mostly hypotonic fluids. Increasing oral fluid intake will lead to a vicious cycle with an increase in fistula or enterostomy output and increased thirst. Therefore, oral intake should be limited to a maximum of 1 L, preferably isotone drinks (at least 50% of intake), and intravenous instead of oral fluid supplementation should be increased in case of thirst or signs of dehydration. The aim is to have a urinary output of at least 1 L a day.^1^

Several studies^10^, ^11^, ^22^, ^23^ show that reconstructive surgery performed in an early phase is associated with higher fistula recurrence rates and mortality. In this cohort, all patients had a “bridging-to-surgery” time of at least 6 months. As our center is the national referral center for type 2 IF, we see patients from all over the country of whom the majority had previous surgical attempts, and therefore, this waiting time of at least 6 months is absolutely recommended. The median time between the last abdominal intervention and reconstructive surgery was 9 months; therefore, most patients needed longer than 6 months to recover to the extent that they could have major surgery. During that period, 66.7% reached the composite endpoint. The other patients could not be optimized any further, for example, because of fistula draining more intra-abdominally than extra-abdominally causing abscesses. In such circumstances, reconstructive surgery was performed after at least 6 months in a shared decision-making process with these patients. Of all patients who completed the 2-year follow-up period and were still alive, 94% percent had reached enteral autonomy (independent on parenteral nutrition or parenteral fluids) anymore. This is comparable with other series in literature^4^, ^12^, ^24^, although we managed the majority of patients at home during PN dependency. The number of catheter-related complications in our cohort was relatively high as expected by the relatively unstable group of patients compared with, for example, type 3 IF patients. The ECF recurrence rate and postoperative mortality are comparable with literature.^23^

A limitation of the present study is the very specific subset of patients studied. Most patients had previous surgical attempts in other hospitals and therefore may represent only the most severe cases and this cohort may not be representative for all type 2 IF patients. Another limitation is the T1 time point measurement used for this study. Due to the waiting list for our IF team outpatient clinic, advice (phone call, e-mail) is given before the first visit to the outpatient clinic. Therefore, optimized treatment was often already started before their first outpatient clinic visit and the T1 study time point might not represent a general population referred for type 2 IF. Unfortunately, for comparison, only reviews^9^, ^25^ and guidelines^1^ are available.

In conclusion, type 2 IF patients can be managed safely at home to recover before undergoing reconstructive surgery. This requires a specialized IF team, a frequent follow**-**up, and tailor-made medical management of nutrition and fluid and electrolyte balances. Sharing knowledge and increasing awareness of the treatment principles in the “bridging-to-surgery” period and prospective registration of all type 2 IF patients will allow for the detection of pitfalls in the “bridging-to-surgery” period, predict outcome, and ultimately improve outcome.

## Electronic Supplementary Material

ESM 1 (DOC 39 kb)

ESM 2 (DOC 48 kb)

ESM 3 (DOC 32 kb)

## References

[1] Vaizey CJ, Maeda Y, Barbosa E (2016). ESCP consensus on the surgical management of intestinal failure in adults. Color Dis..

[2] Visschers RGJ, Olde Damink SWM, Winkens BRGJ (2008). Treatment strategies in 135 consecutive patients with enterocutaneous fistulas. World J Surg.

[3] Slater NJ, Bokkerink WJV, Konijn V (2014). Safety and Durability of 1-Stage Repair of Abdominal Wall Defects With Enteric Fistulas. Ann Surg..

[4] Owen RM, Love TP, Perez SD (2013). Definitive Surgical Treatment of Enterocutaneous Fistula: Outcomes of a 23-Year Experience. JAMA Surg..

[5] von Elm E, Altman DG, Egger M (2014). The strengthening the reporting of observational studies in epidemiology (STROBE) statement: Guidelines for reporting observational studies. Int J Surg..

[6] Kaushal M, Carlson GL (2004). Management of Enterocutaneous Fistulas. Clin Colon Rectal Surg..

[7] Atema JJ, Mirck B, Van Arum I (2016). Outcome of acute intestinal failure. Br J Surg..

[8] de Vries FEE, Reeskamp LF, van Ruler O (2017). Systematic review: pharmacotherapy for high-output enterostomies or enteral fistulas. Aliment Pharmacol Ther..

[9] Lal S, Teubner A, Shaffer JL (2006). Review article: intestinal failure. Aliment Pharmacol Ther..

[10] Lynch AC, Delaney CP, Senagore AJ (2004). Clinical outcome and factors predictive of recurrence after enterocutaneous fistula surgery. Ann Surg..

[11] Hollington P, Bassett P, Windsor AJ (2008). An analysis of predictive factors for healing and mortality in patients with enterocutaneous fistulas. Aliment Pharmacol Ther..

[12] Engledow A, Chan S, Forbes A (2010). The management of enterocutaneous fistula in a regional unit in the United kingdom: a prospective study. Dis Colon Rectum..

[13] Connolly PT, Teubner A, Lees NP (2008). Outcome of reconstructive surgery for intestinal fistula in the open abdomen. Ann Surg..

[14] Creditor MC (1993). Hazards of hospitalization of the elderly. Ann Intern Med..

[15] Hollington P, Mawdsley J, Lim W (2004). An 11-year experience of enterocutaneous fistula. Br J Surg..

[16] Buchman AL, Moukarzel AA, Bhuta S (1995). Parenteral nutrition is associated with intestinal morphologic and functional changes in humans. Jpen J Parenter Enter Nutr..

[17] Hernandez G, Velasco N, Wainstein C (1999). Gut mucosal atrophy after a short enteral fasting period in critically ill patients. J Crit Care..

[18] Bharadwaj S, Ginoya S, Tandon P (2016). Malnutrition: laboratory markers vs nutritional assessment. Gastroenterol Rep..

[19] Karas PL, Goh SL, Dhital K (2015). Is low serum albumin associated with postoperative complications in patients undergoing cardiac surgery?. Interact Cardiovasc Thorac Surg..

[20] Levitt DG, Levitt MD (2016). Human serum albumin homeostasis : a new look at the roles of synthesis, catabolism, renal and gastrointestinal excretion, and the clinical value of serum albumin measurements. Int J Gen Med..

[21] Truong A, Hanna MH, Moghadamyeghaneh Z (2016). Implications of preoperative hypoalbuminemia in colorectal surgery. World J Gastrointest Surg..

[22] Visschers RG, van Gemert WG, Winkens B (2012). Guided treatment improves outcome of patients with enterocutaneous fistulas. World J Surg..

[23] de Vries FEE, Atema JJ, van Ruler O, et al. A Systematic Review and Meta-analysis of Timing and Outcome of Intestinal Failure Surgery in Patients with Enteric Fistula. World J Surg. 2017 (epub ahead of print). 10.1007/s00268-017-4224-z PMC5801381 28924879

[24] Ravindran P, Ansari N, Young CJ (2014). Definitive surgical closure of enterocutaneous fistula: Outcome and factors predictive of increased postoperative morbidity. Colorectal Dis..

[25] Carlson GL, Dark P (2010). Acute intestinal failure. Curr Opin Crit Care..

