# Supplementary material for: Bridging-to-Surgery in Patients with Type 2 Intestinal Failure
Source: J Gastrointest Surg. 2020 Jul 22;25(6):1545–55. doi: 10.1007/s11605-020-04741-0 (PMC8203517; doi:10.1007/s11605-020-04741-0)
Supplement: Supplementary file 1 — (DOC 39 kb) [file 11605_2020_4741_MOESM1_ESM.doc]

**Figure S1. Algorithm for standard care (de Vries et al. 2017^8^)**
