# Supplementary material for: Bridging-to-Surgery in Patients with Type 2 Intestinal Failure
Source: J Gastrointest Surg. 2020 Jul 22;25(6):1545–55. doi: 10.1007/s11605-020-04741-0 (PMC8203517; doi:10.1007/s11605-020-04741-0)
Supplement: Supplementary file 2 — (DOC 48 kb) [file 11605_2020_4741_MOESM2_ESM.doc]

**Figure S2. Flow chart of patient selection**

^Parenteral Nutrition
