# Supplementary material for: Bridging-to-Surgery in Patients with Type 2 Intestinal Failure
Source: J Gastrointest Surg. 2020 Jul 22;25(6):1545–55. doi: 10.1007/s11605-020-04741-0 (PMC8203517; doi:10.1007/s11605-020-04741-0)
Supplement: Supplementary file 3 — (DOC 32 kb) [file 11605_2020_4741_MOESM3_ESM.doc]

**Text S1. Intestinal Failure treatment strategy (updated from Atema et al. BJS, 2016^7^)**

Type 2 intestinal failure (IF) patients are difficult to manage as they have no stable disease yet. Type 2 IF patients can still be catabolic, metabolically unstable and usually have complex abdominal wounds and fistula. Our approach may differ from most other centers in that we manage type 2 IF patients at home, thereby reducing admission time after the most acute infectious problems are managed. In our strategy, patients generally are not hospitalized until reconstruction surgery or cessation of PN. Management in home care setting reduces the pressure for unwanted shortening of the necessary ‘bridging to surgery’ time.

The specific treatment differed between patients, but in general management was as follows. Any signs of residual sepsis were treated aggressively, preferably with percutaneous drainage and antibiotic treatment. To reduce overall output and gastrointestinal secretion and to slow gut transit, a short bowel diet, fluid restriction, antimotility drugs, proton pump inhibitors and, on occasion, somatostatin analogues were prescribed. Teduglutide was not used in any of these patients. In the Netherlands Teduglutide is not reimbursed and not used for type 2 IF patients. Enteral intake was desirable but whether this was possible depended on the length of remaining bowel, the anatomical location of fistulas and stomas, and the effect on manageable fistula output.

In general nil by mouth was neither necessary nor desirable, and trophic feeding was recommended. Some patients were additionally fed by fistuloclysis, if the anatomy was suitable. Patients were given the advice to use a starch enrich but monosaccharides limited diet. PN was administered at home by trained nurses, and prepared and delivered by a specialized pharmacy. Patients could be trained in self-administration of PN and central venous catheter care. The use of tunneled single-lumen central venous catheters (CVC) in the jugular or subclavian vein or a peripherally inserted central catheter was preferred. Catheters were locked with taurolidine and all care of CVC was carried out following a strict aseptic protocol. The central venous catheter was removed only in the event of refractory or ongoing sepsis, or blood cultures positive for fungi or yeasts or highly virulent bacteria. Patients visited the IF outpatient clinic, organized as a ‘one-stop’ multidisciplinary consultation, frequently at 4-, 8- or 12-week intervals. At those visits, the surgeon, internist-endocrinologist, physician assistant or specialized IF nurse and dietitian all evaluated the patient and discussed multidisciplinary general wellbeing/performance status, nutritional state, lab results, PN and/or fluid administration, medication, fistula or stoma output, inspection of fistula/wound/CVC (if applicable) and rehabilitation status. Based on the outcome of these items, a treatment plan was made for the upcoming period. The surgeon planned and performed the reconstructive surgery and ordered additional tests and/or consultations if needed for that (e.g. imaging or plastic surgeon) and oversaw wound/fistula care together with a specialized stoma/wound nurse. The internist-endocrinologist monitored nutritional state, lab results, stoma/fistula output and administration of the amount of parenteral nutrition and fluids as well as CVC-related topics such as managing CVC-related infections or preventive or therapeutic anticoagulation. Specialized stoma/wound nurses addressed fistula/wound and/or stoma. If an enteroatmospheric fistula could be isolated it was managed by use of a fistula adapter and negative-pressure wound therapy (100 mmHg). If isolation was not achievable, gauzes were applied, covered by a wound manager; smaller surfaces were covered by a stoma bag. In between such visits, patients had contact once or twice a month or on indication with the specialized IF nurse/physician assistant by e-mail or telephone in which medication, CVC, stoma/fistula output and urine production was discussed. Twice a month, patients had contact with a dietitian to monitor weight, oral intake and amount of administered PN and/or fluids. The specialized home care teams were instructed to follow the treatment plan and contacted the IF team directly if deemed necessary. After achieving a stable clinical condition and good nutritional status, surgical repair of persistent fistulas and/or restoration of intestinal continuity was considered. Postponing reconstructive surgery for at least 6 months after the last laparotomy or drainage of abdominal abscesses was the rule of thumb. Oral and enema contrast studies or MRI enterography were used to assess the entire length of the small intestine and colon. CT was used to estimate the size and anatomy of the abdominal wall defect. Plastic/reconstructive surgeons were consulted in IF cases of large full-thickness skin defects or in patients with abdominal wall defect associated with significant loss of domain and need of abdominoplasty; these reconstructions are a joined effort of the plastic surgeon and IF surgeon*.* Surgical procedures were based on restoring intestinal continuity and closing the abdomen by reconstruction of the abdominal wall. Fistulating segments of bowel were resected, with restoration of continuity by anastomosis, on occasion with construction of a defunctioning ileostomy. The use of non-absorbable synthetic meshes was avoided. Component separation techniques and non-cross-linked biological mesh (Strattice^TM^ Reconstructive Tissue Matrix; LifeCell, Branchburg, New Jersey, USA) were used for abdominal wall reconstruction. No nasogastric, nasoduodenal or surgical percutaneous feeding tubes were placed for postoperative enteral nutrition, because PN was continued after surgery with stepwise increase of the oral intake until adaptation of intestinal function was restored after weeks to months, sometimes even years*.* Outpatient monitoring was continued for at least 6 months or until PN had been discontinued successfully.
